# Supplementary material for: Skin Rejuvenation Efficacy and Safety Evaluation of Kaempferia parviflora Standardized Extract (BG100) in Human 3D Skin Models and Clinical Trial
Source: Biomolecules. 2024 Jun 29;14(7):776. doi: 10.3390/biom14070776 (PMC11274994; doi:10.3390/biom14070776)
Supplement: Supplementary file 1 [file biomolecules-14-00776-s001.zip › biomolecules-3018026-supplementary.pdf]

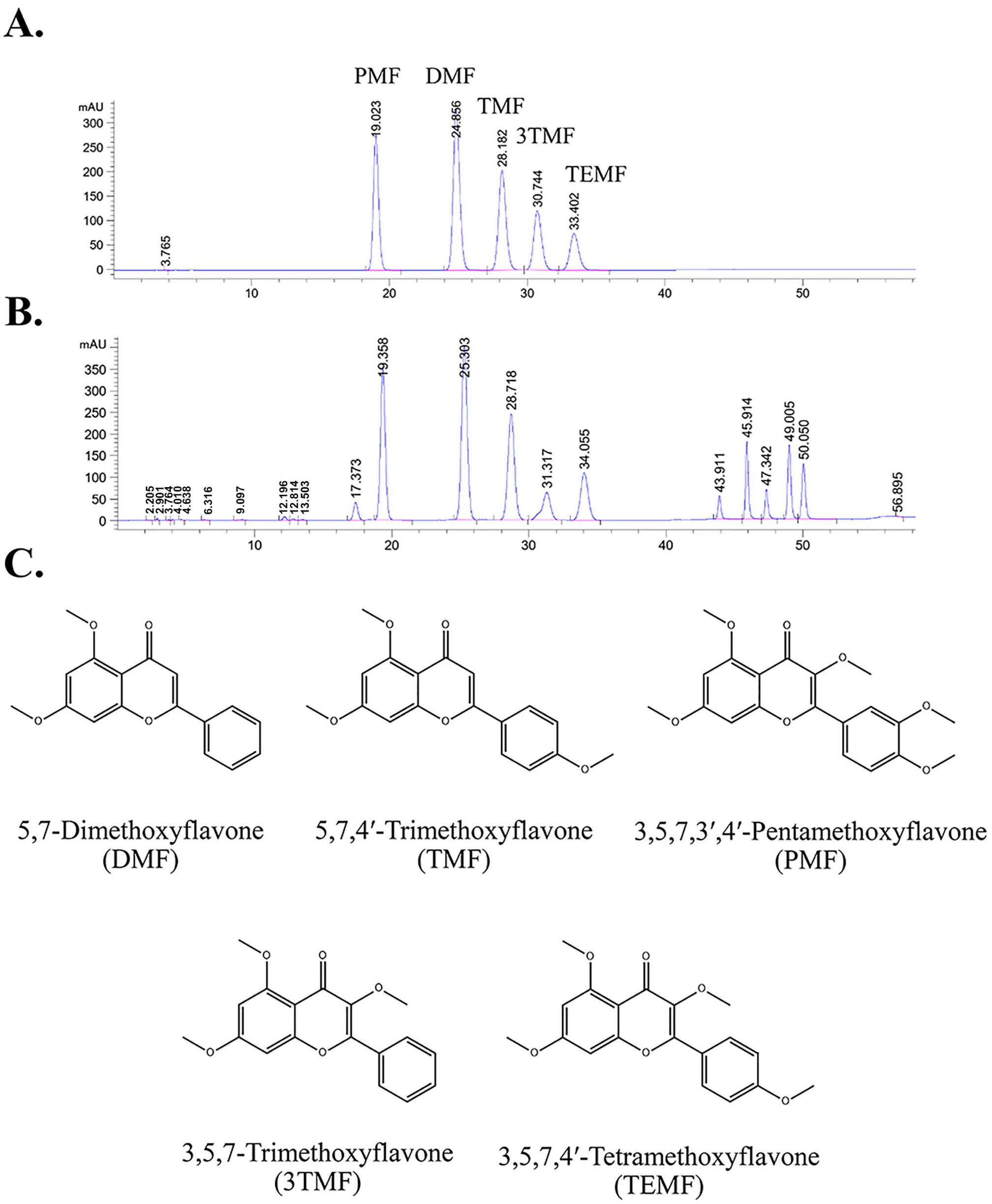

Figure S1. HPLC chromatograms and structures of polymethoxyflavones. (**a**) Representative HPLC chromatograms (UV 254 nm) of standard methoxyflavones, including dimethoxyflavone (DMF), trimethoxyflavone (TMF), polymethoxyflavone (PMF), 3,3',4',5,6,7-hexamethoxyflavone (3TMF), and tetramethoxyflavone (TEMF), at a concentration of 250 ppm. (**b**) Representative HPLC chromatogram (UV 254 nm) of BG100 extract at a concentration of 2,000 ppm. (**c**) Structures of DMF, TMF, PMF, 3TMF, and TEMF.

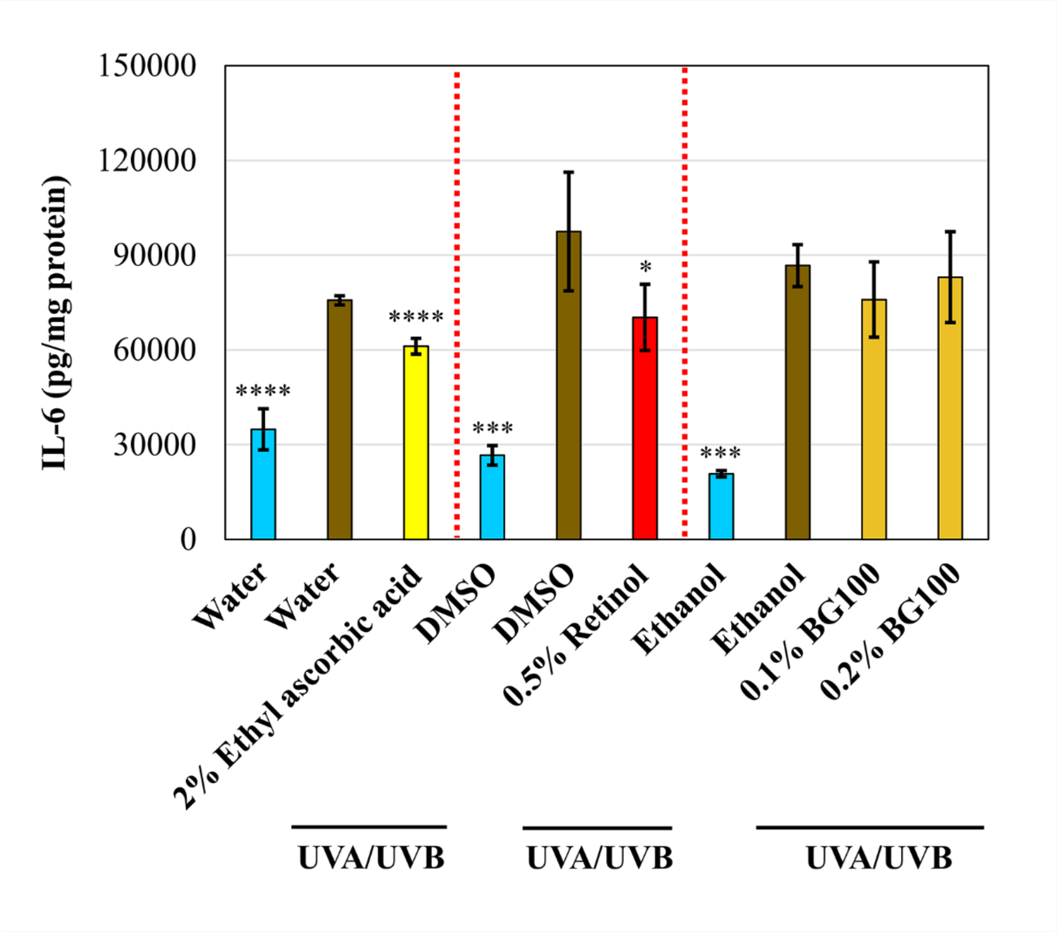

Figure S2. Effects of BG100 extract on IL-6 secretion in UV-exposed human full-thickness 3D skin tissues. IL-6 secretion was measured in the culture media of human full-thickness 3D skin tissues exposed or not exposed to UVA and UVB radiation for 5 days and topically treated with different formulations for 6 days. The treatments included 2% ethyl ascorbic acid in water, water, 0.5% retinol in DMSO, 0.5% DMSO, 0.1% and 0.2% BG100 extract in ethanol, or 1% ethanol. The data were derived from duplicate measurements on two skin tissues for each treatment. Statistical significance was denoted as *$P < 0.05$; ***$P < 0.001$; ****$P < 0.0001$, in comparison to the UV-exposed negative control.

Table S1. Viability of V79-4 cells treated with BG100 extract for 4 and 24 hours.

Treatment	% Cell viability of V79-4 cells (Mean ± SEM)	
	4 hours	24 hours
Negative control	100	100
50 µg/ml BG100 extract	91.82 ± 1.03	89.47 ± 0.23
100 µg/ml BG100 extract	94.34 ± 0.50	81.68 ± 0.19
200 µg/ml BG100 extract	94.65 ± 0.56	67.62 ± 0.37
400 µg/ml BG100 extract	91.82 ± 1.03	45.29 ± 0.37

The cell viability percentage of BG100 extract in V79-4 cells was presented relative to the negative control (culture medium). The data were derived from a single independent experiment, with triplicate measurements.

Table S2. Schedule of the skin sensitization testing in clinical study.

<table>
<tr><th colspan="9">The Induction phase (Week 1-3)</th></tr>
<tr><th colspan="9">Week 1</th></tr>
<tr><td colspan="2">Day of the week</td><td>Mon</td><td>Tue</td><td>Wed</td><td>Thurs</td><td>Fri</td><td>Sat</td><td>Sun</td></tr>
<tr><td colspan="2">Study day</td><td>D0</td><td>D1</td><td>D2</td><td>D3</td><td>D4</td><td>D5</td><td>D6</td></tr>
<tr><td colspan="2">Product application</td><td>√</td><td></td><td>√</td><td></td><td>√</td><td></td><td></td></tr>
<tr><td colspan="2">Removal/Assessment (A)</td><td></td><td></td><td>√</td><td></td><td>√</td><td></td><td></td></tr>
<tr><th colspan="9">Week 2</th></tr>
<tr><td colspan="2">Day of the week</td><td>Mon</td><td>Tue</td><td>Wed</td><td>Thurs</td><td>Fri</td><td>Sat</td><td>Sun</td></tr>
<tr><td colspan="2">Study day</td><td>D7</td><td>D8</td><td>D9</td><td>D10</td><td>D11</td><td>D12</td><td>D13</td></tr>
<tr><td colspan="2">Product application</td><td>√</td><td></td><td>√</td><td></td><td>√</td><td></td><td></td></tr>
<tr><td colspan="2">Removal/Assessment (A)</td><td>√</td><td></td><td>√</td><td></td><td>√</td><td></td><td></td></tr>
<tr><th colspan="9">Week 3</th></tr>
<tr><td>Day of the week</td><td>Mon</td><td>Tue</td><td>Wed</td><td>Thurs</td><td>Fri</td><td>Sat</td><td>Sun</td><td>Mon</td></tr>
<tr><td>Study day</td><td>D14</td><td>D15</td><td>D16</td><td>D17</td><td>D18</td><td>D19</td><td>D20</td><td>D21</td></tr>
<tr><td>Product application</td><td>√</td><td></td><td>√</td><td></td><td>√</td><td></td><td></td><td></td></tr>
<tr><td>Removal/Assessment (A)</td><td>√</td><td></td><td>√</td><td></td><td>√</td><td></td><td></td><td>√</td></tr>
<tr><th colspan="9">The Resting phase (Week 4-5)</th></tr>
<tr><th colspan="9">The Challenge phase (Week 6)</th></tr>
<tr><td colspan="4">Day of the week</td><td>Mon</td><td>Tue</td><td>Wed</td><td>Thurs</td><td>Fri</td></tr>
<tr><td colspan="4">Study day</td><td>D35</td><td>D36</td><td>D37</td><td>D38</td><td>D39</td></tr>
<tr><td colspan="4">Product application</td><td>√</td><td></td><td></td><td></td><td></td></tr>
<tr><td colspan="4">Removal/Assessment (A)</td><td></td><td></td><td>√</td><td></td><td>√ (A)</td></tr>
</table>

Table S3. Characteristics of study participants.

Subject	Age	Sex	Phototype	Comment	Inclusion date	End date
1	60	F	IV	None	Sep 26, 2022	Nov 4, 2022
2	54	F	IV	None	Sep 26, 2022	Nov 4, 2022
3	49	F	III	None	Sep 26, 2022	Nov 4, 2022
4	56	F	IV	None	Sep 26, 2022	Nov 4, 2022
5	60	F	III	None	Sep 26, 2022	Nov 4, 2022
6	56	F	IV	None	Sep 26, 2022	Nov 4, 2022
7	31	F	IV	None	Sep 26, 2022	Nov 4, 2022
8	35	F	IV	None	Sep 26, 2022	Nov 4, 2022
9	49	F	IV	None	Sep 26, 2022	Nov 4, 2022
10	46	F	IV	None	Sep 26, 2022	Nov 4, 2022
11	35	F	IV	None	Sep 26, 2022	Nov 4, 2022
12	54	F	IV	None	Sep 26, 2022	Nov 4, 2022
13	51	F	IV	None	Sep 26, 2022	Nov 4, 2022
14	43	F	IV	None	Sep 26, 2022	Nov 4, 2022
15	34	F	IV	None	Sep 26, 2022	Nov 4, 2022
16	44	F	IV	None	Sep 26, 2022	Nov 4, 2022
17	60	F	IV	None	Sep 26, 2022	Nov 4, 2022
18	45	F	IV	None	Sep 26, 2022	Nov 4, 2022
19	54	F	IV	None	Sep 26, 2022	Nov 4, 2022

20	59	F	IV	None	Sep 26, 2022	Nov 4, 2022
21	45	F	IV	None	Sep 26, 2022	Nov 4, 2022
22	46	F	IV	None	Sep 26, 2022	Nov 4, 2022
23	59	F	IV	None	Sep 26, 2022	Nov 4, 2022
24	36	F	IV	None	Sep 26, 2022	Nov 4, 2022
25	53	F	IV	None	Sep 26, 2022	Nov 4, 2022
26	52	F	IV	None	Sep 26, 2022	Nov 4, 2022
27	57	F	IV	None	Sep 26, 2022	Nov 4, 2022
28	52	F	IV	None	Sep 26, 2022	Nov 4, 2022
29	56	F	IV	None	Sep 26, 2022	Nov 4, 2022
30	46	F	III	None	Sep 26, 2022	Nov 4, 2022
31	49	M	IV	None	Sep 26, 2022	Nov 4, 2022
32	39	F	IV	None	Sep 26, 2022	Nov 4, 2022
33	24	F	IV	None	Sep 26, 2022	Nov 4, 2022
34	23	M	IV	None	Sep 26, 2022	Nov 4, 2022
35	38	F	IV	None	Sep 26, 2022	Nov 4, 2022
36	46	M	IV	None	Sep 26, 2022	Nov 4, 2022
37	44	F	IV	None	Sep 26, 2022	Nov 4, 2022
(38)[*]	*(41)*[*]	*(F)*[*]	*(IV)*[*]	*(Major:non-adherence)*[*]	*Sep 26, 2022*[*]	*Oct 9, 2022*[*]
39	57	F	IV	None	Sep 26, 2022	Nov 4, 2022
40	42	F	IV	None	Sep 26, 2022	Nov 4, 2022
41	39	F	IV	None	Sep 26, 2022	Nov 4, 2022

42	26	F	IV	None	Sep 26, 2022	Nov 4, 2022
*(43)**	*(33)**	*(F)**	*(IV)**	*(Major:non-adherence)**	*Sep 26, 2022**	*Oct 17, 2022**
44	55	F	IV	None	Sep 26, 2022	Nov 4, 2022
45	54	F	IV	None	Sep 26, 2022	Nov 4, 2022
46	51	F	III	None	Sep 26, 2022	Nov 4, 2022
47	56	F	IV	None	Sep 26, 2022	Nov 4, 2022
48	47	F	IV	None	Sep 26, 2022	Nov 4, 2022
49	58	F	IV	None	Sep 26, 2022	Nov 4, 2022
50	58	M	IV	None	Sep 26, 2022	Nov 4, 2022
51	58	F	IV	None	Sep 26, 2022	Nov 4, 2022
52	52	F	IV	None	Sep 26, 2022	Nov 4, 2022
53	56	F	IV	None	Sep 26, 2022	Nov 4, 2022
54	45	F	IV	None	Sep 26, 2022	Nov 4, 2022

Female is abbreviated as F, and Male is abbreviated as M. Values enclosed in parentheses with an asterisk indicate data points excluded from the analysis.

Table S4. Description of non-adherence in the clinical study.

Description of the non-adherence	Type of non-adherence	Data kept into the analysis
Subject #38 did not come on D7 and withdrew the consent on D13	Major	No
Subject #43 withdrew the consent on D21	Major	No
Subject #46 did not come on D7	Minor	Yes

Table S5. Medical and surgical treatments before and during the study.

**Subject**	**Medication**	**Indication**	**Beginning of treatment** (compared to the kinetics)	**End of treatment** (compared to the kinetics)
3	Paracetamol	Headache	D0	D0
14	Paracetamol	Headache	D7	D7
48	Paracetamol	Headache	D18	D18

Table S6. Irritation potential of 0.2% BG100 extract during the induction phase in healthy human volunteers.

Score	Day 2		Day 4		Day 7		Day 9		Day 11		Day 14		Day 16		Day 18		Day 21	
	N	%	N	%	N	%	N	%	N	%	N	%	N	%	N	%	N	%
T+	10	19.2%	5	9.6%	3	5.8%	0	0.0%	0	0.0%	0	0.0%	0	0.0%	0	0.0%	0	0.0%
0	34	65.4%	35	67.3%	44	84.6%	41	78.8%	43	82.7%	36	69.2%	49	94.2%	49	94.2%	47	90.4%
0.5	8	15.4%	12	23.1%	5	9.6%	11	21.2%	9	17.3%	16	30.8%	3	5.8%	3	5.8%	5	9.6%
1	0	0.0%	0	0.0%	0	0.0%	0	0.0%	0	0.0%	0	0.0%	0	0.0%	0	0.0%	0	0.0%
1.5	0	0.0%	0	0.0%	0	0.0%	0	0.0%	0	0.0%	0	0.0%	0	0.0%	0	0.0%	0	0.0%
2	0	0.0%	0	0.0%	0	0.0%	0	0.0%	0	0.0%	0	0.0%	0	0.0%	0	0.0%	0	0.0%
2.5	0	0.0%	0	0.0%	0	0.0%	0	0.0%	0	0.0%	0	0.0%	0	0.0%	0	0.0%	0	0.0%
3	0	0.0%	0	0.0%	0	0.0%	0	0.0%	0	0.0%	0	0.0%	0	0.0%	0	0.0%	0	0.0%
4	0	0.0%	0	0.0%	0	0.0%	0	0.0%	0	0.0%	0	0.0%	0	0.0%	0	0.0%	0	0.0%
5	0	0.0%	0	0.0%	0	0.0%	0	0.0%	0	0.0%	0	0.0%	0	0.0%	0	0.0%	0	0.0%
6	0	0.0%	0	0.0%	0	0.0%	0	0.0%	0	0.0%	0	0.0%	0	0.0%	0	0.0%	0	0.0%

T+: Reactions to the negative control and corresponding readings were excluded from the data analysis. N represents the number of subjects, and % indicates the percentage of subjects.
